# *Sema6B*, *Sema6C*, and *Sema6D* Expression and Function during Mammalian Retinal Development

**DOI:** 10.1371/journal.pone.0063207

**Published:** 2013-04-30

**Authors:** Ryota L. Matsuoka, Lu O. Sun, Kei-ichi Katayama, Yutaka Yoshida, Alex L. Kolodkin

**Affiliations:** 1 Howard Hughes Medical Institute, The Solomon H. Snyder Department of Neuroscience, The Johns Hopkins University School of Medicine, Baltimore, Maryland, United States of America; 2 Division of Developmental Biology, Cincinnati Children's Hospital Medical Center, Cincinnati, Ohio, United States of America; School of Biomedical Sciences, The University of Queensland, Australia

## Abstract

In the vertebrate retina, the formation of neural circuits within discrete laminae is critical for the establishment of retinal visual function. Precise formation of retinal circuits requires the coordinated actions of adhesive and repulsive molecules, including repulsive transmembrane semaphorins (Sema6A, Sema5A, and Sema5B). These semaphorins signal through different Plexin A (PlexA) receptors, thereby regulating distinct aspects of retinal circuit assembly. Here, we investigate the physiological roles of three Class 6 transmembrane semaphorins (Sema6B, Sema6C, and Sema6D), previously identified as PlexA receptor ligands in non-retinal tissues, in mammalian retinal development. We performed expression analysis and also phenotypic analyses of mice that carry null mutations in each of genes encoding these proteins using a broad range of inner and outer retinal markers. We find that these Class 6 semaphorins are uniquely expressed throughout postnatal retinal development in specific domains and cell types of the developing retina. However, we do not observe defects in stereotypical lamina-specific neurite stratification of retinal neuron subtypes in *Sema6B^−/−^* or *Sema6C^−/−^; Sema6D^−/−^* retinas. These findings indicate these Class 6 transmembrane semaphorins are unlikely to serve as major PlexA receptor ligands for the assembly of murine retinal circuit laminar organization.

## Introduction

Nervous system function relies in part on precise patterns of synaptic connectivity established during development. In the vertebrate retina, external visual information is processed by distinct subtypes of retinal neurons that elaborate and establish synaptic connections within discrete laminae: the outer plexiform layer (OPL) and sublaminae of the inner plexiform layer (IPL). Within the IPL, two parallel neural circuit pathways respond to either an increment (ON pathway) or a decrement (OFF pathway) in illumination, and these are organized within separate sublaminae to allow for segregated processing of distinct visual stimuli. However, the molecular mechanisms that govern specific neuronal subtype targeting to retinal laminae in the inner and outer retina remain poorly understood.

Accumulating evidence shows that both adhesive and repulsive molecules direct neurite targeting to laminae in the inner and outer retina *in vivo*. Cell adhesion molecules, including sidekicks and Dscams, disturb lamina-specific neurite arborization within the IPL in chick and mouse [1], [2], [3]. In addition, multiple transmembrane semaphorins, including Sema6A, Sema5A, and Sema5B, regulate neurite targeting of many cell types in the inner and outer murine retina [4], [5], [6]. Specifically, Sema6A signals through PlexA4 to regulate select neuronal subtype targeting to specific sublaminae within the IPL [6]. Sema6A-PlexA4 signaling also controls horizontal cell neurite arborization, neurite targeting to the OPL, and rod ribbon synapse formation in the outer retina [5]. Conversely, Sema5A and Sema5B signal through PlexA1 and PlexA3 to prevent neurites of multiple inner retinal neuron subtypes from aberrantly misprojecting into the outer retina, thereby constraining these neurites within the inner retina [4]. Taken together, these findings demonstrate that the PlexA receptors play critical roles in the formation of retinal circuits. However, it is not known whether PlexA receptor ligands previously identified in non-retinal tissues also contribute to the retinal circuit formation.

The class 6 transmembrane semaphorins Sema6B, Sema6C, and Sema6D also signal through the PlexA receptors. For example, Sema6B binds to PlexA4, inducing inhibitory responses in hippocampal and sympathetic neurons that are PlexA4-dependent *in vitro* [7], [8]. In contrast, Sema6C and Sema6D bind to PlexA1, regulating specific aspects of neuronal and cardiac development [9], [10]. These findings raise the question as to whether or not Sema6B-PlexA4 signaling, or Sema6C- and/or Sema6D-PlexA1 signaling, control retinal development. Therefore, we here investigate whether Sema6B, Sema6C, and Sema6D regulate retinal laminar organization and circuit formation.

## Materials and Methods

### Animals

The day of birth in this study is designated as postnatal (P) day 0. The *Sema6B^−/−^*, *Sema6C^−/−^*, and *Sema6D^−/−^* mice were previously described [8], [11], [12]. For the phenotypic assessment of adult wild-type, *Sema6B^−/−^*, and *Sema6C^−/−^; Sema6D^−/−^* retinas, 4 independent animals of each genotype were analyzed. For the phenotypic assessment of P17 wild-type, *Sema6B^−/−^*, and *Sema6C^−/−^; Sema6D^−/−^* retinas, 2 independent animals of each genotype were analyzed. This study was carried out in strict accordance with the recommendations in the Guide for the Care and Use of Laboratory Animals of the National Institutes of Health. The protocol was approved by the Animal Care and Use Committee of the Johns Hopkins University School of Medicine (Protocol Number: M011M80). Mice were euthanized prior to tissue harvesting to minimize suffering.

### Immunohistochemistry

Eyes were fixed in 4% paraformaldehyde for 1 hr at 4°C, equilibrated in 30% sucrose/PBS and embedded in OCT embedding media (Tissue-Tek). Retinal sections (20–40 µm) were blocked in 5% fetal bovine serum in 1 X PBS and 0.4% Triton-X100 for 1 hr at room temperature and then incubated overnight at 4°C with primary antibodies: rabbit anti-tyrosine hydroxylase (Millipore at 1∶1000), goat anti-ChAT (Millipore at 1∶100), rabbit anti-calretinin (Swant at 1∶2500), guinea pig anti-vGlut3 (Millipore at 1∶2500), rabbit anti-Dab-1 (generous gift from Dr. Brian Howell at 1∶500), rabbit anti-calbindin (Swant at 1∶2500), rabbit anti-N-terminal melanopsin (ATS at 1∶2000), rabbit anti-PlexA2 (generous gift from Dr. Fumikazu Suto at 1∶400) [13], Armenian hamster anti-PlexA4 (generous gift from Dr. Fumikazu Suto at 1∶400) [13], mouse anti-PKCα (Millipore at 1∶200), mouse anti-synaptotagmin 2 (ZNP-1, Zebrafish International Resource Center at 1∶2000), rabbit anti-neurokinin 3 receptor (Calbiochem at 1∶3000), rabbit anti-cone arrestin (generous gift from Dr. Cheryl Craft at 1∶3000), guinea pig anti-vGlut1 (Millipore at 1∶2000), chicken anti-vimentin (Millipore at 1∶1000), and mouse anti-glutamine synthetase (Millipore at 1∶1000). Sections were washed 6 times for 5 min in 1 X PBS and then incubated with secondary antibodies and TO-PRO-3 (Molecular Probe at 1∶400) for 1 hr at room temperature. Sections were washed 6 times for 5 min in PBS and coverslips were mounted using vectorshield hard set fluorescence mounting medium (Vector laboratories), and confocal fluorescence images were taken using a Zeiss Axioskop2 Mot Plus, LSM 5 pascal confocal microscope.

### *In situ* Hybridization

*In situ* hybridization was performed on fresh frozen retina sections (20 µm thickness) as described previously [6]. The digoxigenin-labeled antisense riboprobes specific for *Sema6B*, *Sema6C*, and *Sema6D* used in this study were previously described [8], [9].

## Results

### *Sema6B*, *Sema6C*, and *Sema6D* mRNA Expression in the Developing Mouse Retina

**Figure 1:**
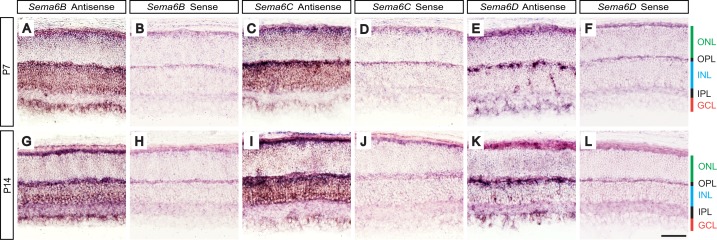
*Sema6B*, *Sema6C*, and *Sema6D* mRNA expression during postnatal retinal development. *In situ* hybridization was performed using antisense probes specific for *Sema6B* (**A**, **G**), *Sema6C* (**C**, **I**), or *Sema6D* (**E**, **K**) on retina sections from P7 (**A**–**F**) and P14 (**G**–**L**) postnatal ages of mice. Adjacent P7 (**B**, **D**, **F**) and P14 (**H**, **J**, **L**) retina sections incubated with sense probes for *Sema6B* (**B**, **H**), *Sema6C* (**D**, **J**), or *Sema6D* (**F**, **L**) were shown as references for background staining. *Sema6B*, *Sema6C*, and *Sema6D* are all expressed at these developmental time points. Strong *Sema6B* expression is found in the GCL and INL at P7 and P14 (**A**, **G**). *Sema6C* is expressed broadly in the INL and sparsely in the GCL (**C**, **I**) at P7 and P14. *Sema6D* is strongly expressed in specific cell types that reside in the GCL and INL close to the OPL at P7 and P14. *Sema6D* expression is also observed sparsely in the inner INL (**E**, **K**). GCL: ganglion cell layer, IPL: inner plexiform layer, INL: inner nuclear layer, OPL: outer plexiform layer, ONL: outer nuclear layer. Scale bar: 50 µm in **L** for **A**–**L**.

To investigate whether Sema6B, Sema6C, and Sema6D regulate retinal development, we first analyzed mRNA expression of *Sema6B*, *Sema6C*, and *Sema6D* during postnatal retinal development by *in situ* hybridization (Fig. 1). We performed *in situ* hybridization at the postnatal ages of P7 and P14, developmental time points when inner and outer retinal circuits are established. We found that *Sema6B*, *Sema6C*, and *Sema6D* are all expressed at these stages of postnatal retinal development. Specifically, *Sema6B* is expressed most prominently in the ganglion cell layer (GCL) and inner nuclear layer (INL) at P7 and P14 (Fig. 1A, 1G). At both ages, a majority of retinal neurons in the GCL and inner INL express *Sema6B* (Fig. 1A, 1G), whereas *Sema6B* expression was either low or absent in the outer nuclear layer (ONL) at these postnatal ages. On the other hand, *Sema6C* was expressed broadly in the INL and sparsely in the GCL at P7 and P14 (Fig. 1C, 1I). A majority of retinal neurons in the INL strongly express *Sema6C*, but only a subset of neurons in the GCL appear to express *Sema6C* at both ages. *Sema6D* expression was strong in neuronal subtypes that reside in the GCL and INL at P7 and P14 (Fig. 1E, 1K). The retinal cell types that express *Sema6D* robustly in the GCL at these ages are retinal ganglion cells and/or displaced amacrine cells, while those expressing *Sema6D* strongly in the INL close to the OPL are likely horizontal cells, given their morphological features and horizontal process staining (Fig. 1E, 1K). At P7 and P14, *Sema6D* was also expressed in a subset of neurons in the inner INL (Fig. 1E, 1K). We used the corresponding sense probes as references for detecting background staining and did not observe specific signals with these probes at P7 and P14 (Fig. 1B, 1D, 1F, 1H, 1J, 1L).

These expression patterns of *Sema6B*, *Sema6C*, and *Sema6D* during postnatal retinal development suggest a role for these semaphorins in regulating retinal development *in vivo*.

### Normal Neurite Stratification of Inner Retinal Neurons in *Sema6B^−/−^* and *Sema6C^−/−^; Sema6D^−/−^* Retinas

To determine whether these class 6 semaphorins regulate retinal development *in vivo*, we analyzed mice that harbour null mutations in genes encoding Sema6B (*Sema6B^−/−^*), or both Sema6C and Sema6D (*Sema6C^−/−^; Sema6D^−/−^*) [8], [12]. For each genotype we analyzed eyes from 4 animals. We examined neurite elaboration in distinct retinal subtypes in the IPL and OPL of these mutant mice by immunohistochemistry using a range of retinal cell-type markers. Each of these markers labels neurites belonging to specific subtypes of RGCs, amacrine and bipolar cells within the IPL or photoreceptors, bipolar and horizontal cells within the OPL.

**Figure 2:**
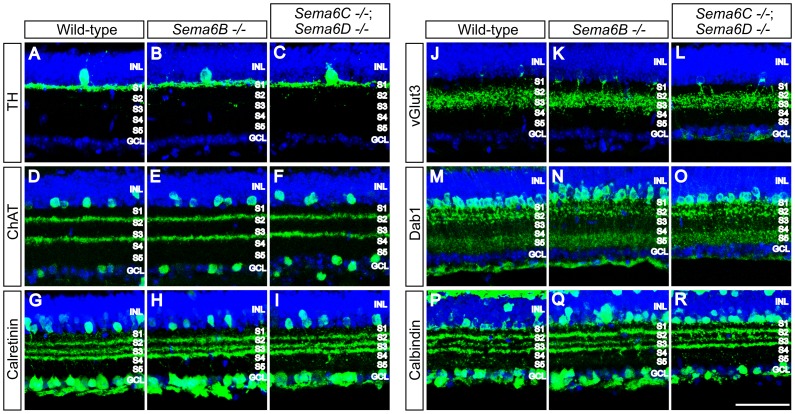
Neurite stratification of amacrine cell and RGC subtypes in the IPL of *Sema6B^−/−^* and *Sema6C^−/−^; Sema6D^−/−^* retinas. Wild-type (**A, D, G, J, M, P**), *Sema6B^−/−^* (**B, E, H, K, N, Q**), and *Sema6C^−/−^*; *Sema6D^−/−^* (**C, F, I, L, O, R**) adult retina sections were immunostained with antibodies against tyrosine hydroxylase (TH) (**A–C**), a marker for dopaminergic amacrine cells, choline acetyltransferase (ChAT) (**D–F**), a marker for cholinergic amacrine cells, calretinin (**G–I**), a marker for subsets of amacrine cells and RGCs, vGlut3 (**J–L**), a marker for subsets of amacrine cells, Dab-1 (**M–O**), a marker for AII amacrine cells, or calbindin (**P–R**), a marker for subsets of amacrine cells and RGCs. TO-PRO-3 dye (blue) was used to visualize the organization of nuclear layers in the retina. The amacrine cell and RGC subtypes labelled by these markers do not show obvious defects in their sublaminar neurite targeting and stratification in *Sema6B^−/−^* or *Sema6C^−/−^*; *Sema6D^−/−^* retinas, as compared to those in wild-type retinas. Scale bar: 50 µm in R for **A–R**.

**Figure 3:**
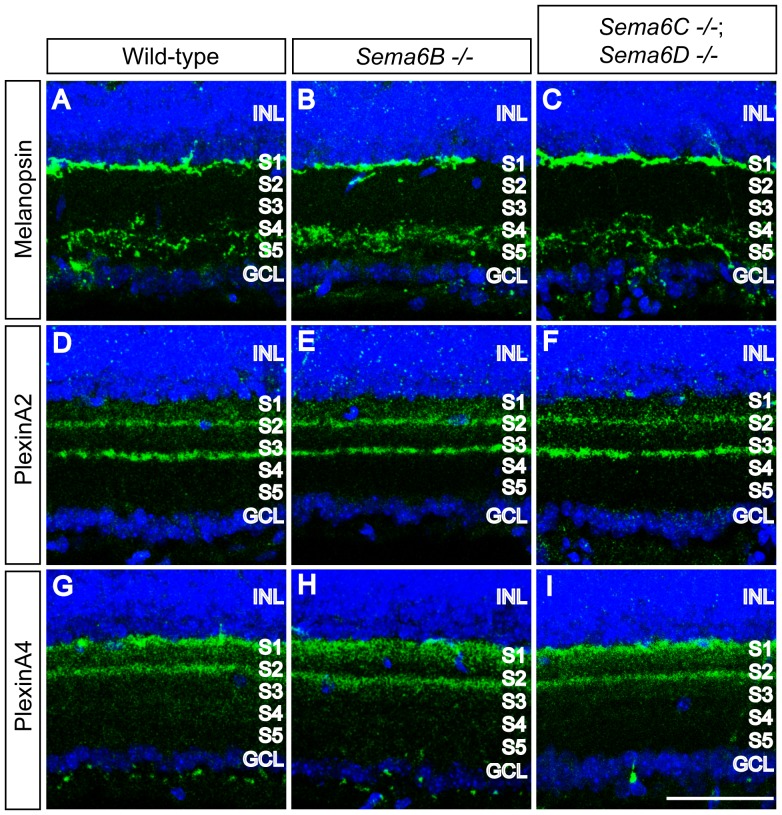
Neurite stratification of ipRGCs, PlexA2-positive, PlexA4-positive cells in the IPL of *Sema6B^−/−^* and *Sema6C^−/−^; Sema6D^−/−^* retinas. (**A–I**) Wild-type (**A, D, G**), *Sema6B^−/−^* (**B, E, H**), and *Sema6C^−/−^*; *Sema6D^−/−^* (**C, F, I**) P17 retina sections were immunostained with antibodies against melanopsin (**A–C**), PlexA2 (**D–F**), or PlexA4 (**G–I**). Retinal neuron subtypes labelled by each of these markers exhibit neurite stratification within specific sublaminae in wild-type retinas, and these sublaminar targeting and stratification patterns are not apparently affected in *Sema6B^−/−^* or *Sema6C^−/−^*; *Sema6D^−/−^* retinas. Scale bar: 50 µm in I for **A–I**.

In adult wild-type retina sections stained with these markers, we observed that multiple amacrine cell subtypes, including dopaminergic, cholinergic, vGlut3^+^, and AII, all exhibited stereotypical lamina-specific neurite stratifications within the IPL (Fig. 2A, 2D, 2J, 2M). None of these subtypes in *Sema6B^−/−^* or *Sema6C^−/−^; Sema6D^−/−^* retinas appeared to differ from those in wild-type retinas (Fig. 2A–2F, 2J–2O). In addition, amacrine cell and RGC subtypes labelled by anti-calbindin or anti-calretinin, both of which define neurite stratification at the borders of S1 and S2, S2 and S3, and S3 and S4 within the IPL of wild-type retinas (Fig. 2G, 2P), exhibit apparently normal neurite stratification in *Sema6B^−/−^* or *Sema6C^−/−^; Sema6D^−/−^* retinas (Fig. 2G–2I, 2P–2R). To investigate whether RGC dendritic stratification is affected in these mutant retinas, we used an antibody directed against the N-terminus of melanopsin that labels multiple subtypes of intrinsically photosensitive retinal ganglion cells (ipRGCs) [14], [15], [16]. In wild-type P17 retinas, ipRGC dendritic arbors are found in two distinct domains of the IPL; one stratifies within the S1 sublamina and the other resides within the S4/S5 sublaminae (Fig. 3A). We found that these ipRGC dendritic stratifications within these two different domains of the IPL do not apparently differ in *Sema6B^−/−^* or *Sema6C^−/−^; Sema6D^−/−^* retinas compared to wild-type retinas (Fig. 3A–3C). We next investigated whether PlexA-positive neurite stratification within the developing IPL is compromised in these mutant retinas. We previously found that PlexA1 and PlexA3 show broad protein expression across the developing postnatal IPL [4], whereas PlexA2 and PlexA4 are localized in specific, but distinct, sublaminae of the developing IPL [5], [6]. To assess whether PlexA2- or PlexA4-positive neurite stratification is affected in these mutant retinas, we labelled P17 retina sections from wild-type, *Sema6B^−/−^*, or *Sema6C^−/−^; Sema6D^−/−^* mice with antibodies specific for PlexA2 or PlexA4 [13]. We observed that PlexA2-positive and also PlexA4-positive neurites stratify in specific sublaminae of wild-type retinas (Fig. 3D, 3G), and that these stratification patterns do not differ in *Sema6B^−/−^* or *Sema6C^−/−^; Sema6D^−/−^* retinas (Fig. 3E, 3F, 3H, 3I).

**Figure 4:**
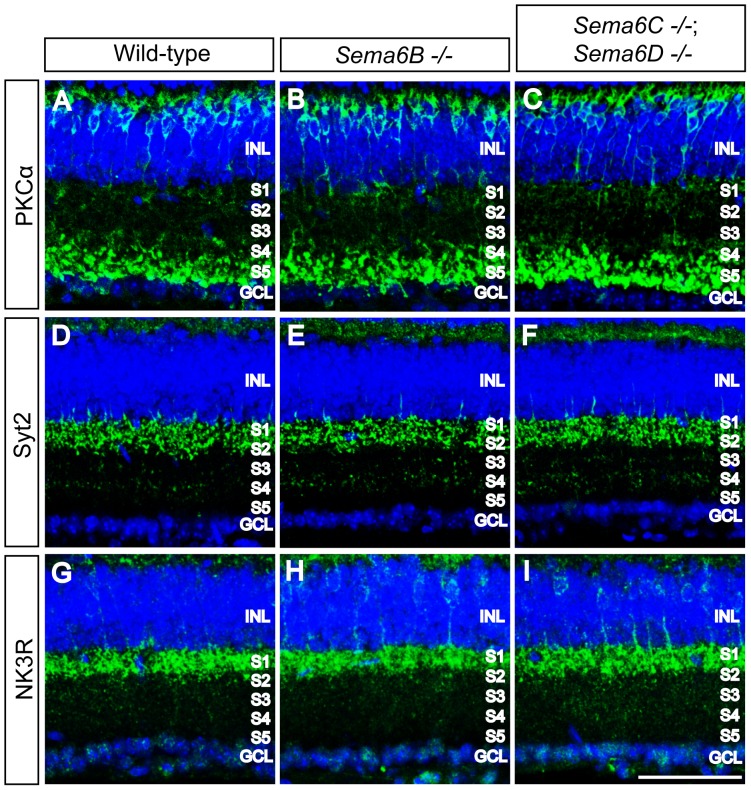
Targeting by distinct subtypes of bipolar cell axons in the IPL of *Sema6B^−/−^* and *Sema6C^−/−^; Sema6D^−/−^* retinas. Wild-type (**A, D, G**), *Sema6B^−/−^* (**B, E, H**), and *Sema6C^−/−^*; *Sema6D^−/−^* (**C, F, I**) adult retina sections were immunostained with antibodies against protein kinase C alpha (PKCα, **A–C**, a marker for rod bipolar cells), synaptotagmin2 (Syt2, D–F, a marker for type-2 cone OFF and type-6 cone ON bipolar cells), and neurokinin-3 receptor (NK3R, **G–I**, a marker for type-5 cone ON and cone OFF bipolar cells). Distinct subtypes of bipolar cells labelled by these three markers elaborate axon terminals within specific sublaminae of wild-type retinas, and these axon termination patterns do not apparently differ in *Sema6B^−/−^* and *Sema6C^−/−^*; *Sema6D^−/−^* retinas. Scale bar: 50 µm in I for **A–I**.

To ask whether bipolar cell axon targeting within specific sublaminae of the IPL is directed by Sema6B, Sema6C, or Sema6D, we used antibodies against protein kinase C alpha (PKCα), synaptotagmin2 (Syt2), or neurokinin-3 receptor (NK3R) to label distinct bipolar cell subtypes [17], [18], [19]: rod bipolar cell axons specifically labelled by anti-PKCα; type2 cone OFF and type6 cone ON bipolar cell axons labelled by anti-Syt2; type-5 cone ON and cone OFF bipolar cell axons labelled by anti-NK3R. All of these processes can be observed to stratify within specific IPL sublaminae of adult wild-type retinas (Fig. 4A, 4D, 4G), and these distinct bipolar cell axon targeting events to specific sublaminae are not apparently affected in *Sema6B^−/−^* or *Sema6C^−/−^; Sema6D^−/−^* retinas (Fig. 4).

### Normal Neurite Stratification of Outer Retinal Neurons in *Sema6B^−/−^* and *Sema6C^−/−^; Sema6D^−/−^* Retinas

**Figure 5:**
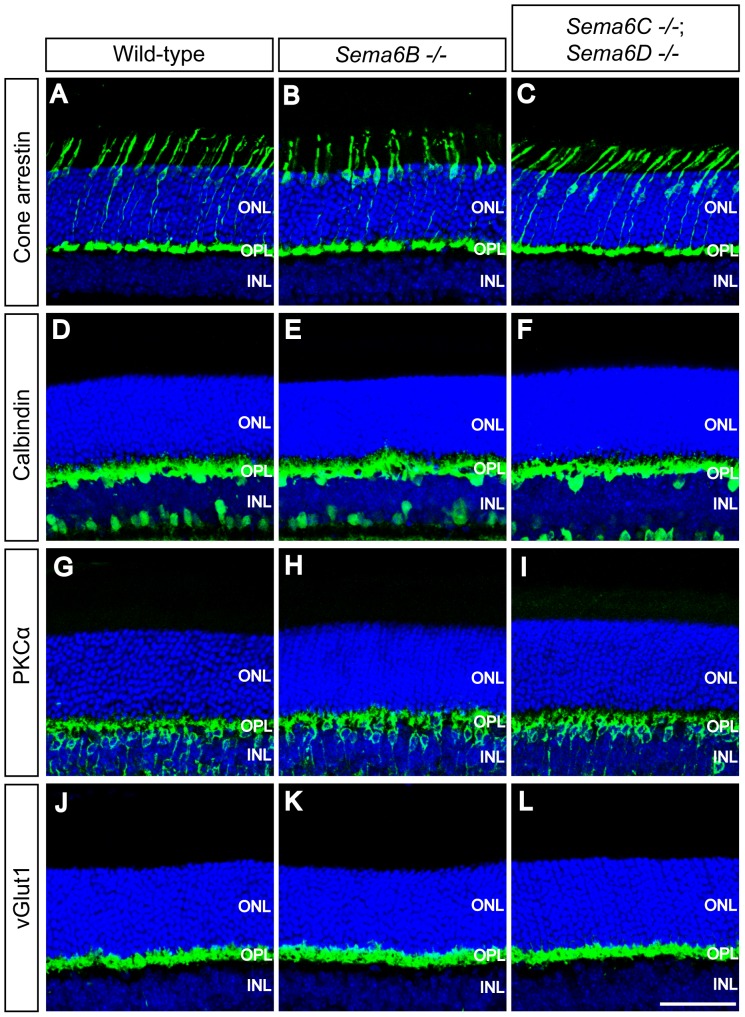
Neurite stratification of outer retinal neuron cell types in the OPL of *Sema6B^−/−^* and *Sema6C^−/−^; Sema6D^−/−^* retinas. Wild-type (**A, D, G, J**), *Sema6B^−/−^* (**B, E, H, K**), and *Sema6C^−/−^*; *Sema6D^−/−^* (**C, F, I, L**) adult retina sections were immunostained with antibodies against cone arrestin (**A–C**, a marker for cone photoreceptors), calbindin (**D–F**, a marker for horizontal cells), PKCα (**G–I**, a marker for rod bipolar cells), and vGlut1 (**J–L**, a marker for photoreceptor axonal terminals). Neurite stratification of photoreceptors, horizontal cells, and bipolar cells visualized by each of these markers does not show any obvious defects in *Sema6B^−/−^* and *Sema6C^−/−^; Sema6D^−/−^* retinas as compared to wild-type retinas. Scale bar: 50 µm in L for **A–L**.

To investigate whether Sema6B, Sema6C, or Sema6D regulate neurite targeting to the OPL, we analyzed neurite stratification of outer retinal neuron cell types; these include horizontal and bipolar cells, and photoreceptors, within the OPL of *Sema6B^−/−^* or *Sema6C^−/−^; Sema6D^−/−^* retinas. We used antibodies directed against calbindin, PKCα, cone arrestin, or vesicular glutamate transport protein 1 (vGlut1) to label horizontal cells, rod bipolar cells, cone photoreceptors, or photoreceptor axonal terminals, respectively [20], [21], [22]. In wild-type adult retinas, these markers reveal horizontal cell neurites, rod bipolar cell dendrites, and cone and rod photoreceptor axon terminals stratifying within the OPL (Fig. 5A, 5D, 5G, 5J), and these neuronal OPL stratification events are not apparently different in *Sema6B^−/−^* and *Sema6C^−/−^; Sema6D^−/−^* retinas (Fig. 5).

### Müller Glia Morphology and Process Extension are Normal in *Sema6B^−/−^* and *Sema6C^−/−^; Sema6D^−/−^* Retinas

**Figure 6:**
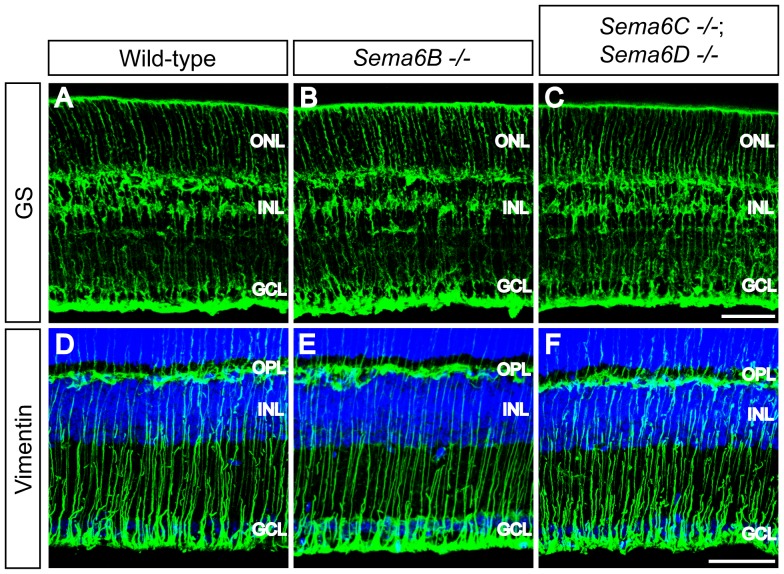
Müller glia cell morphology in *Sema6B^−/−^* and *Sema6C^−/−^; Sema6D^−/−^* retinas. Wild-type (**A**, **D**), *Sema6B^−/−^* (**B**, **E**), and *Sema6C^−/−^*; *Sema6D^−/−^* (**C**, **F**) adult retina sections were immunostained with antibodies against glutamine synthetase (GS, **A**–**C**) or vimentin (**D**–**F**). Overall morphology, process extension, and cell body position of Müller glia cells visualized by these two markers do not differ among wild-type, *Sema6B^−/−^*, and *Sema6C^−/−^*; *Sema6D^−/−^* retinas. Scale bar: 50 µm in **C** for **A**–**C**, and in **F** for **D**–**F**.

To assess whether morphology and process extension of Müller glia cells are affected in *Sema6B^−/−^* and *Sema6C^−/−^; Sema6D^−/−^* retinas, we used antibodies directed against glutamine synthetase and vimentin to visualize cell bodies and processes of Müller glia cells. Müller glia cell bodies are found in the INL, and their cell processes span across the retina and form the outer and inner limiting membranes in wild-type retinas (Fig. 6A, 6D) [23]. We observed that the morphology of Müller glia cells in adult retinas from *Sema6B^−/−^* and *Sema6C^−/−^; Sema6D^−/−^* mice does not apparently differ from that in wild-type retinas (Fig. 6).

## Discussion

We show here that the formation of neural circuitry in the inner and outer retina of *Sema6B^−/−^* and *Sema6C^−/−^; Sema6D^−/−^* mice is comparable to that observed in WT mice. Previous studies show that these class 6 transmembrane semaphorins signal through the PlexA receptors to regulate neural and non-neural development [7], [8], [9], [10]. We recently found that Sema6A and several PlexA receptors play critical roles in regulating the development of retinal circuits [4], [5], [6], and thus we hypothesized that these class 6 transmembrane semaphorins might function through PlexA receptors to regulate retinal development.

Our results show that these class 6 transmembrane semaphorins are all expressed in specific and unique retinal cell types during postnatal development. To investigate the possibility that these transmembrane proteins regulate retinal development, we analyzed *Sema6B^−/−^* and *Sema6C^−/−^; Sema6D^−/−^* mutant retinas by immunohistochemistry using a range of markers that label distinct retinal neuron subtypes in the inner and outer retina. However, our extensive phenotypic analyses of these mutant retinas did not reveal clear deficits in stereotypical neurite stratification of distinct subtypes in either the inner or outer retina. Although we used many immunological markers that label different subtypes of retinal cells, our analysis did not include several additional retinal neuron subtypes present in the retina. We do not exclude the possibility that more specific retinal neuronal or glial subtypes, which were not visualized by the markers used here, show defects in neurite elaboration or sublaminar targeting. It is also possible that penetrance of retinal phenotypes, if any, in these mutant mice is not high enough to be revealed by the number of the mutant mice we examined (n = 4 mice/genotype analyzed). We also did not assess the cell number and cell migration of each retinal subtype, nor did we investigate mosaic patterns of cell body distribution of these subtypes in the horizontal retinal plane. Finally, our analysis did not include a detailed analysis of retinal neuronal or glial process morphology in the plane of individual laminae. Thus, our present study does not exclude the possibility of phenotypes in *Sema6B^−/−^* and *Sema6C^−/−^; Sema6D^−/−^* retinas that affect these aspects of retinal organization. Nevertheless, given the important roles played by PlexA receptors in sublaminar targeting of multiple retinal neuron subtypes [4], [5], [6], our work shows that these class 6 semaphorins do not serve major functions in retinal sublaminar targeting. Since the transmembrane semaphorins Sema5A and Sema5B together constrain neurites from inner retinal neuron subtypes to the IPL [4], it is possible that class6 semaphorins are functionally redundant with respect to regulation of retinal development. It was shown that Sema6A and Sema6B function additively to regulate murine hippocampal mossy fiber projections *in vivo* [8], and thus Sema6B might also cooperate with Sema6A to control specific events of retinal development, such as horizontal cell OPL neurite stratification.

We expect that a variety of membrane-bound and secreted guidance cues will be investigated further to identify the complete spectrum of molecules required for the establishment of laminar organization of the vertebrate retina. Recent studies show that secreted guidance cues, including netrins and slits, direct lamina-specific photoreceptor and RGC axon targeting in the fly and zebrafish visual system [24], [25]. It will be of interest to determine how membrane-bound and secreted cues cooperate to build the laminar structure of vertebrate retinal circuits, advancing our understanding of how segregated processing of visual information in the retina comes to be established during development.
